# Indispensable role of the Ubiquitin-fold modifier 1-specific E3 ligase in maintaining intestinal homeostasis and controlling gut inflammation

**DOI:** 10.1038/s41421-018-0070-x

**Published:** 2019-01-29

**Authors:** Yafei Cai, Guangxun Zhu, Siyang Liu, Zezheng Pan, Michaela Quintero, Candace J. Poole, Chunwan Lu, Huabin Zhu, Bianca Islam, Jan van Riggelen, Darren Browning, Kebin Liu, Richard Blumberg, Nagendra Singh, Honglin Li

**Affiliations:** 1 College of Animal Science and Technology, Nanjing Agricultural University, 210095 Nanjing, Jiangsu China; 2 Department of Biochemistry & Molecular Biology, Georgia Cancer Center, Medical College of Georgia, Augusta University, Augusta, GA 30912 USA; 3 Department of Stomatology, Tongji Hospital, Tongji Medical College, Huazhong University of Science and Technology, 430030 Wuhan, Hubei China; 4 Department of Biochemistry, Faculty of Basic Medicine, Nanchang University, 330006 Nanchang, Jiangxi China; 5 Division of Gastroenterology, Department of Medicine, Brigham and Women’s Hospital, Harvard Medical School, Boston, MA 02115 USA; 6 The 10th People’s Hospital, Tongji University, Shanghai, China

**Keywords:** Developmental biology, Cell signalling

## Abstract

Intestinal exocrine secretory cells, including Paneth and goblet cells, have a pivotal role in intestinal barrier function and mucosal immunity. Dysfunction of these cells may lead to the pathogenesis of human diseases such as inflammatory bowel disease (IBD). Therefore, identification and elucidation of key molecular mechanisms that regulate the development and function of these exocrine cells would be crucial for understanding of disease pathogenesis and discovery of new therapeutic targets. The Ufm1 conjugation system is a novel ubiquitin-like modification system that consists of Ufm1 (Ubiquitin modifier 1), Uba5 (Ufm1-activating enzyme, E1), Ufc1 (Ufm1-conjugating enzyme, E2) and poorly characterized Ufm1 E3 ligase(s). Recent mouse genetic studies have demonstrated its indispensable role in embryonic development and hematopoiesis. Yet its role in other tissues and organs remains poorly defined. In this study, we found that both Ufl1 and Ufbp1, two key components of the Ufm1 E3 ligase, were highly expressed in the intestinal exocrine cells. Ablation of either Ufl1 and Ufbp1 led to significant loss of both Paneth and goblet cells, which in turn resulted in dysbiotic microbiota and increased susceptibility to experimentally induced colitis. At the cellular and molecular levels, *Ufbp1* deficiency caused elevation of endoplasmic reticulum stress and activation of the Unfolded Protein Response (UPR) and cell death program. Administration of small molecular chaperone partially prevented loss of Paneth cells caused by acute Ufbp1 deletion. Taken together, our results have provided unambiguous evidence for the crucial role of the Ufm1 E3 ligase in maintenance of intestinal homeostasis and protection from inflammatory diseases.

## Introduction

The intestinal epithelium is composed of a single layer of epithelial cells that are rapidly renewed through proliferation and differentiation of intestinal stem cells (ISCs). In addition to digestion and absorption of nutrients, the intestinal epithelium also serves as a defensive barrier to prevent against infection of gut pathogens. Intestinal epithelial cells (IECs) consist of stem cells, proliferating progenitor cells and highly differentiated absorptive and secretory cells. Whereas absorptive enterocytes are responsible for nutrient absorption, professional secretory cells such as Paneth and goblet cells have a crucial role in maintaining intestinal homeostasis and mucosal immunity. Goblet cells synthesize and secrete mucin proteins, the major component of intestinal mucus that protects the epithelium from bacterial infection^1^. Paneth cells are responsible for synthesis and secretion of a large quantity of antimicrobial peptides that maintain homeostatic microbiota in the gut^2,3^. They also have a crucial role in the formation of ISC niche and regulation of self-renewal and differentiation of ISCs under conditions such as calorie restriction^4,5^. A breakdown of intestinal tissue homeostasis may lead to human diseases such as inflammatory bowel disease (IBD). Loss of Paneth cells or impairment of their normal function is often observed in the IBD patients and contributes to disease pathogenesis^6,7^. Goblet cell deficiency or dysfunction is also causative for ulcerative colitis (UC)^8,9^. Therefore, elucidation of the underlying mechanism that governs development, survival and function of Paneth and goblet cells would be crucial for development of novel therapies for inflammatory diseases.

Post-translational modification of target proteins by Ubiquitin (Ub) and Ubiquitin-like (Ubl) modifiers has important roles in multiple cellular processes, and dysfunction of this process has been implicated in the pathogenesis of a variety of human diseases^10^. Ufm1 (Ubiquitin-fold modifier 1) is a small Ubl modifier with 85 amino acid residues and highly conserved in multi-cellular organisms^11^. With a limited primary sequence identity with Ub and other Ubls, Ufm1 displays a solution structure of ubiquitin-fold that is shared by other Ubls^12^. However, unlike Ub and some Ubls with di-glycine (GG) residues at the carboxyl (C)-terminus of their active forms, the active Ufm1 contains Valine-Glycine (VG) residues at its C-terminus, and its Glycine residue is covalently conjugated to the lysine residues of target proteins via an iso-peptide bond. Ufm1 conjugation to its target proteins, a process termed as ufmylation, is accomplished in multi-step biochemical reactions that are catalyzed by a set of Ufm1-specific enzymes, namely, Ufm1-activating E1 enzyme Uba5, Ufm1-conjugating E2 enzyme Ufc1, and Ufm1-specific E3 ligase(s)^11^. However, the identity of Ufm1 targets and the mechanism to control this conjugating process is poorly understood.

Although Ufm1-sepcific E1 (Uba5) and E2 (Ufc1) enzymes share signature domains with other E1s and E2s, only one Ufm1-specific E3 ligase has been identified so far and this E3 ligase does not contain any conventional E3 ligase domains or motifs that mediate ubiquitination or ubiquitination-like modifications. Ufl1 (Ufm1 ligase 1, as known as KIAA0776, RCAD, NLBP and Maxer) was originally isolated by several independent studies as a novel Cdk5rap3-binding protein and regulator of NF-κB signaling^13–15^. Interestingly, Tatsumi et al. found that Ufl1 promoted ufmylation of Ufbp1 (Ufm1 binding protein 1, also named as DDRGK1, C20orf116 and Dashurin), an endoplasmic reticulum (ER)-anchored Ufl1-binding protein^14,16,17^. This study provided biochemical evidence for the notion that Ufl1 may function as a Ufm1-specific E3 ligase. Yoo et al. subsequently found that Ufl1 also promoted ufmylation of ASC1 (Activating Signal Cointegrator 1, also known as TRIP4), a transcription co-activator of nuclear receptors^18,19^. In breast cancer cells, ufmylated ASC1 serves as a platform to facilitate recruitment of estrogen receptor and other transcription co-activators and enhance transcription of target genes, thereby promoting breast cancer development^19^. Interestingly, ufmylated Ufbp1 functions as a co-factor in the process of ASC1 ufmylation^19^.

Since its discovery more than a decade ago, the physiological function of the Ufm1 conjugation system has remained unclear until very recently. Genetic studies using gene knockout (KO) mouse models have provided undisputable evidence for its essential role in animal development. Germline deletion of individual ufmylation genes, including *Uba5*, *Ufl1* and *Ufbp1*, led to impaired red blood cell development and embryonic lethality around E11.5–E13.5 days^20–22^. Acute ablation of either *Ufl1* or *Ufbp1* in adult mice caused pancytopenia and severe anemia, further highlighting the indispensable role of ufmyation in erythropoiesis^20–22^. In addition to its role in animal development, multiple lines of evidence indicate involvement of the ufmylation pathway in the pathogenesis of various human dieseases, including hematopoietic diseases^23,24^, diabetes^25^, ischemic heart injury^26^, skeletal dysplasia^27^, atherosclerosis^28^, and cancer^19,29^. Intriguingly, variants of human *Ufm1*, *Uba5 and Ufc1 genes* have been found to be linked to early-onset encephalopathy and defective brain development^30–35^, while human *Ufbp1* was identified as one of the new genetic risk loci in Parkinson’s disease^36^, indicating a crucial role of ufmylation in neural development and function. Furthermore, loss-of-function mutation in *Ufbp1* gene was found to be a causative factor in Shohat-type spondyloepimetaphyseal dysplasia (SEMD), a skeletal dysplasia that affects cartilage development^37^. Mutations in Ufm1-specific protease Ufsp2 were found in the patients with Beukes hip dysplasia^27^. Taken together, these recent findings underscore the importance of the Ufm1 system in normal development and physiology. Nonetheless, the full scope of its biological function and involvement in disease pathogenesis remains to be further defined.

During the course of characterizing *Ufl1* and *Ufbp1* conditional knockout mice, we frequently observed gut bleeding in some conditional knockout mice, and this observation prompted us to investigate the potential role of these genes in the intestine. Using both acute and tissue-specific knockout mouse models, we found that ablation of either *Ufl1* or *Ufbp1* led to significant loss of Paneth and goblet cells, which in turn resulted in dysbiotic microbiota and increased susceptibility to experimentally induced colitis. Our findings have identified the ufmylation pathway as a novel molecular mechanism to control intestinal homeostasis.

## Results

### Acute ablation of *Ufl1* caused significant loss of Paneth and goblet cells in the intestine

**Fig. 1:**
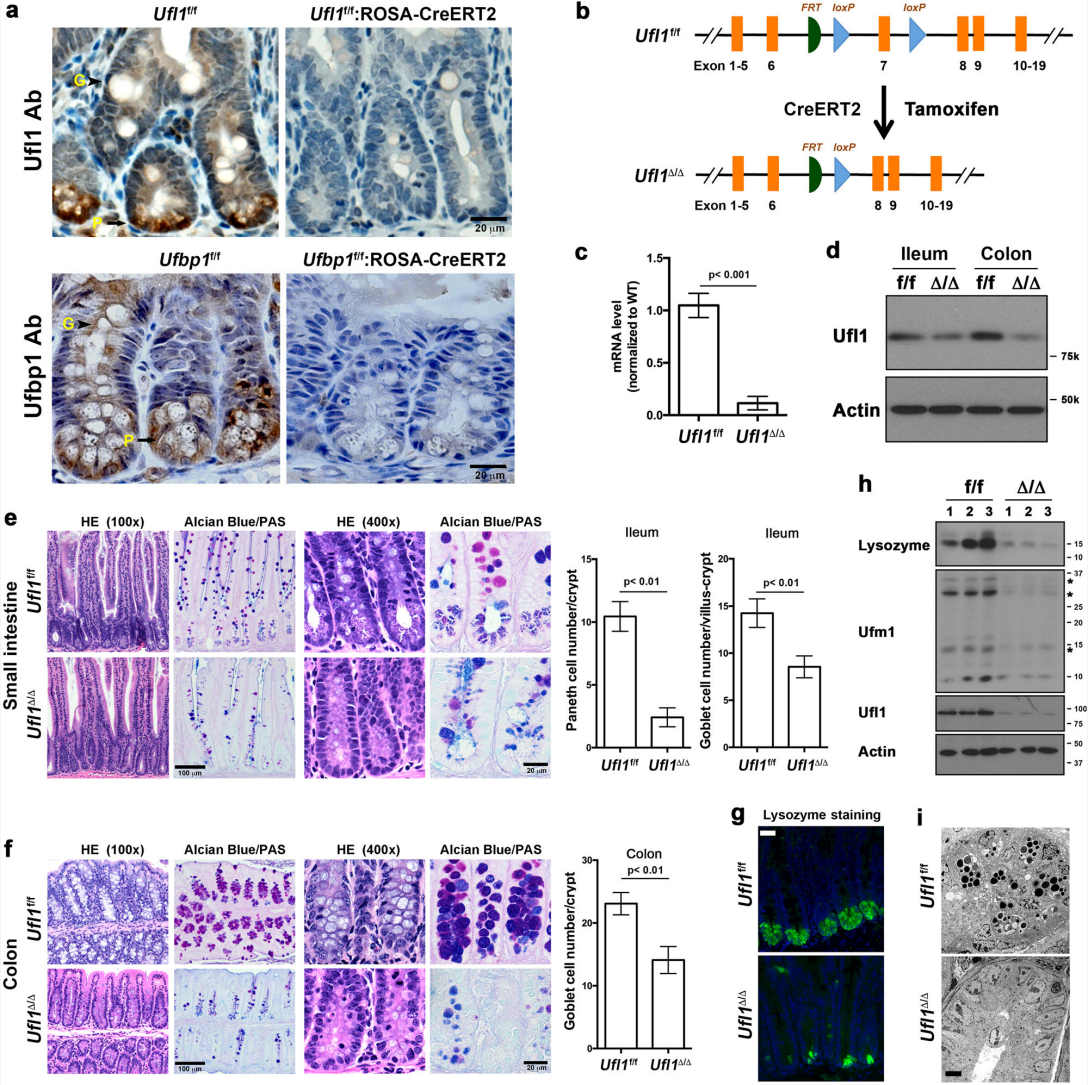
*Ufl1* deficiency led to significant loss of Paneth and goblet cells in the intestine. **a** Ufl1 and Ufbp1 are highly expressed in intestinal Paneth cells. Ufl1 and Ufbp1 proteins were examined by immunohistochemistry of ileal sections. *Ufl1*^f/f^:ROSA26-CreERT2 and *Ufbp1*^f/f^: ROSA26-CreERT2 mice were treated with tamoxifen for 5 days and killed at 2 weeks after initial treatment of tamoxifen. Goblet cells are labeled with “G”, while Paneth cells are marked with “P”. **b** Tamoxifen-induced deletion of *Ufl1* exon 7. **c**. *Ufl1* mRNA level in *Ufl1*-deficient intestine. Quantitative RT-PCR was performed using total RNA isolated from small intestinal crypts and the primers specific for deleted exon 7. The *p* values were determined by unpaired *t*-tests. *p* < 0.001 (*n* = 6). **d** Ufl1 protein level in wild-type and *Ufl1*-deleted intestines. **e** H&E and Alcian/PAS staining of small intestinal sections and quantitation of Paneth and goblet cells in the crypts. At least 100 crypts were counted from 6 mice of each genotype in a double blinded manner. The average numbers of Paneth and goblet cells per crypt were scored. The *p* values were determined by unpaired *t*-tests. **f** H&E and Alcian/PAS staining of the colonic sections and quantitation of goblet cells in the crypts (*n* = 6). Quantitation was conducted as described in “**e**”. **g** Lysozyme staining of ileal sections. **h** Immunoblotting of lysozyme in *Ufl1-*deficient crypts. Crypts were isolated accordingly and the lysates were subjected to immunoblotting of indicated antibodies. Ufmylation conjugates were indicated by asterisks. Scale bar is 20 μm. **i** Electron micrographs of the crypts of wild-type and *Ufl1*-deficient mice. Scale bar is 5 μm

We first examined expression of Ufl1 and Ufbp1 in the IECs. Both Ufl1 and Ufbp1 are present in all types of IECs, and highly expressed in exocrine Paneth cells (Fig. 1a). Antibody specificity was confirmed by lack of staining in IECs with tamoxifen-induced deletion of each gene, respectively (Fig. 1a). To investigate the function of Ufl1 in the intestine, we acutely ablated *Ufl1* in adult *Ufl1* floxed mice (*Ufl1*^f/f^:ROSA26-CreERT2) by tamoxifen administration (Fig. 1b). Deletion of *Ufl1* allele in the gut was confirmed by quantitative RT-PCR using primers specific for the deleted exon 7 (Fig. 1c), immunoblotting of Ufl1 protein (Fig. 1d, h) and immunohistochemistry (Fig. 1a). Although the gross anatomy of the intestinal epithelium was not compromised in *Ufl1*-deleted mice, close examination of intestinal sections revealed substantial loss of both Paneth and goblet cells in the crypts of *Ufl1*-deficient ileum (Fig. 1e) and goblet cells in the colon (Fig. 1f). The result was further confirmed by Alcian blue/Periodic acid–Schiff (PAS) staining (Fig.1e, f), which specifically marks secretory cells such as Paneth and goblet cells. Loss of Paneth cells in the small intestine was also confirmed by reduced staining of lysozyme, an early Paneth cell-specific marker (Fig. 1g). Decreased amount of lysozyme in *Ufl1*-deficient intestine was further demonstrated by immunoblotting of lysozyme in crypt lysates (Fig. 1h). To exclude the possibility that *Ufl1* deficiency may impair lysozyme synthesis which may mimic Paneth cell loss, we performed electron microscopy (EM) of the crypts. In comparison to wild-type intestinal crypts that contained many Paneth cells with extensive ER network and secretory granules, *Ufl1*-deficient crypts had very few cells with a rich ER network, indicating the absence of Paneth cells (Fig. 1i). Taken together, these results strongly suggest that Ufl1 is crucial for intestinal exocrine cells.

### IEC-specific knockout of Ufbp1 led to depletion of Paneth and goblet cells

**Fig. 2:**
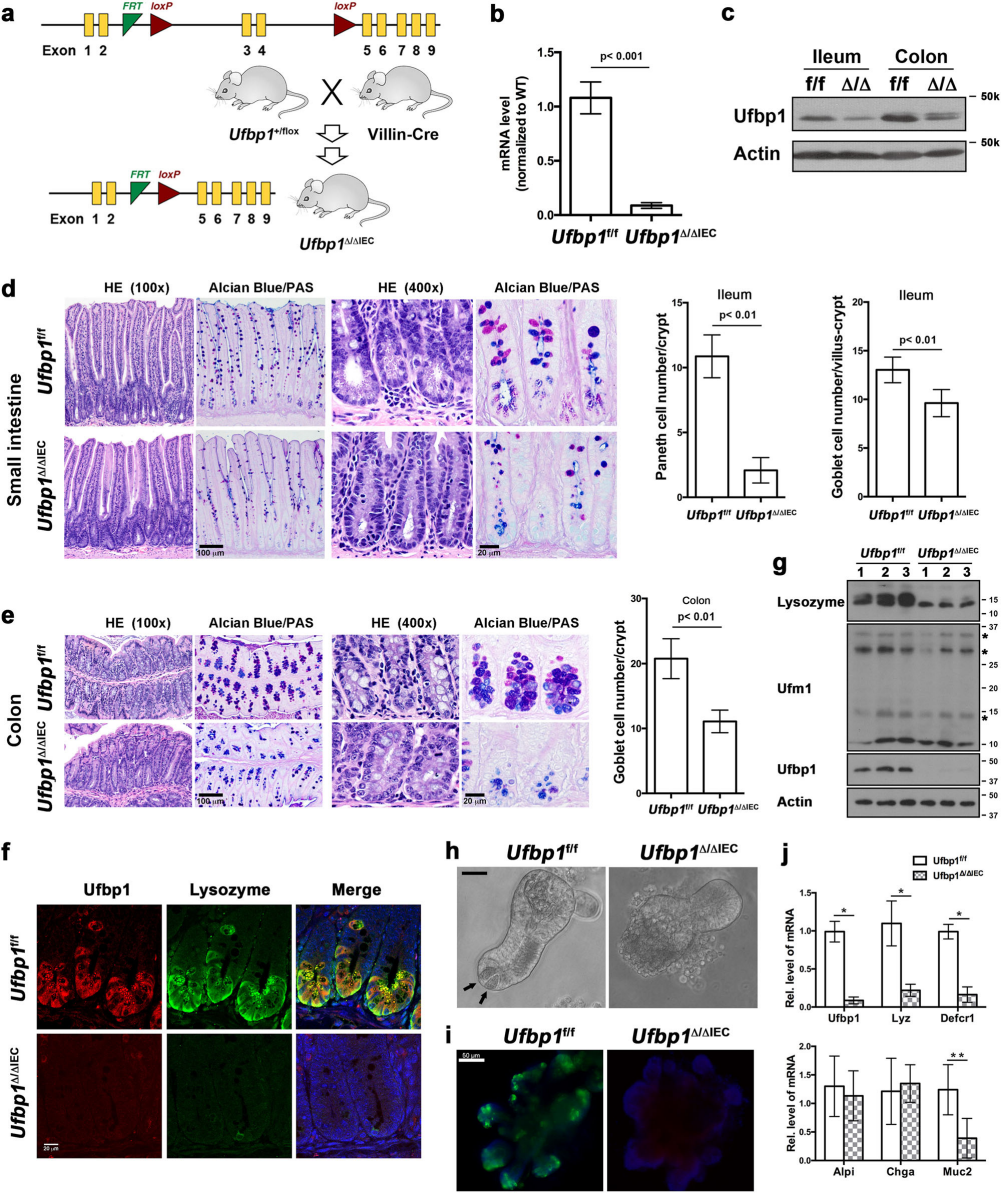
IEC-specific deletion of *Ufbp1* resulted in depletion of Paneth and goblet cells. **a** *Ufbp1* floxed allele and generation of IEC-specific *Ufbp1* knockout mice. **b** *Ufbp1* mRNA level in *Ufbp1-*deficient intestine. Quantitative RT-PCR was performed using total small intestinal crypt RNA and the primers specific for deleted exons 3 and 4. The *p* values were determined by unpaired *t*-tests *p* < 0.001 (*n* = 6). **c** Ufbp1 protein level in *Ufbp1*^∆/∆IEC^ intestine. **d** H&E and Alcian/PAS staining of the small intestinal sections and quantitation of Paneth and goblet cells in *Ufbp1*^∆/∆IEC^ mice. At least 100 crypts were counted from 6 mice of each genotype in a double blinded manner. The average numbers of Paneth and goblet cells per crypt were scored. The *p* values were determined by unpaired *t*-tests. **e** H&E and Alcian/PAS staining of the colonic sections and quantitation of goblet cells in the crypts (*n* = 6). **f** Double immunostaining of ileal sections using Ufbp1 and lysozyme antibodies. **g** Immunoblotting of lysozyme in *Ufl1-*deficient crypts. Crypts were isolated accordingly and the lysates were subjected to immunoblotting of indicated antibodies. Ufmylation conjugates were indicated by asterisks. **h** In vitro organoid culture of wild-type and *Ufbp1-*deficient crypts. Paneth cells in the wild-type organoid were marked by arrows. Scale bar is 50 μm. **i** Lysozyme immunostaining of cultured organoid. **j** Quantitative RT-PCR analysis of gene expression using total RNA isolated from culture organoids. Six independent cultures for each genotype were used for the analysis. The *p* values were determined by unpaired *t*-tests. *p < 0.001, **p < 0.01 (*n* = 6). *Lyz* lysozyme; *Defcr1* defensin α1; *Alpi* alkaline phosphatase; *Chga* Chromogranin A; and *Muc2* mucin 2

In addition to its putative role as a Ufm1-specific E3 ligase, Ufl1 is also involved in regulating the stability of its interacting proteins, C53 and Ufbp1, in a Ufm1-independent manner^14,16^. Ufbp1 is a Ufm1 substrate and a co-factor of Ufm1 E3 ligase^16,19^, and *Ufbp1* null mice exhibit the phenotype similar to the one of *Ufl1* knockout mice with defective embyronic development and impaired hematopoiesis^21,22^. To further identify Ufl1’s downstream effector and define their functional role in the gut, we therefore generated *Ufbp1* IEC-specific knockout model using Villin-Cre transgenic mice (Fig. 2a)^38^. Deletion of exons 3 and 4 resulted in loss of Ufbp1 protein in the intestine (Fig. 2b, c, g). Similar to *Ufl1* conditional KO mice, IEC-specific deletion of *Ufbp1* (*Ufbp1*^∆/∆IEC^) led to a significant loss of Paneth and goblet cells in the intestinal epithelium (Fig. 2d–f). Paneth cell loss was further confirmed by decreased expression of Paneth cell-specific genes, such as lysozyme (Fig. 2g), Defcr1 and 5 (Supplementary Fig. S1a). Interestingly, the number of enteroneuroendocrine cells (marked by Chromogranin A) was not affected by *Ufbp1* deficiency (Supplementary Fig. S1b). Although isolated *Ufbp1*-deficient crypts were able to grow and develop into mature organoids (Fig. 2h), these cultured organoids did not contain Paneth cells and their absence was further validated by lack of lysozyme staining and reduction of Paneth cell-specific gene expression (Fig. 2h–j). Together, our results demonstrate that like Ufl1, Ufbp1 is also essential for intestinal exocrine cells.

### Paneth cell-specific deletion of *Ufbp1* resulted in the decrease of Paneth cells

**Fig. 3:**
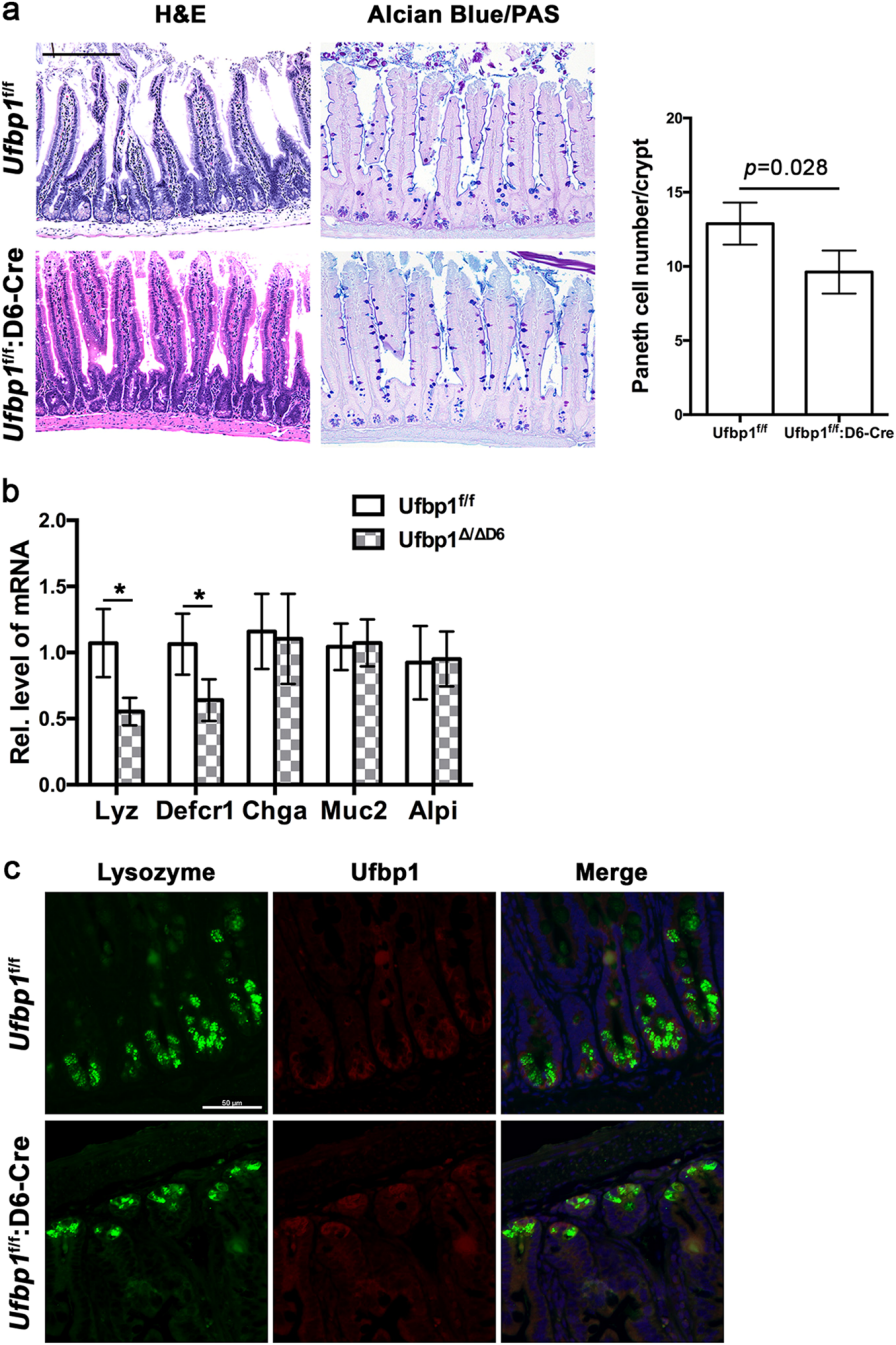
Paneth cell-specific ablation of *Ufbp1* led to the decrease of Paneth cells. **a** H&E and Alcian/PAS staining of small intestinal sections and quantitation of Paneth cells in the crypts. At least 100 crypts were counted from 6 mice of each genotype in a double blinded manner. The average numbers of Paneth cells per crypt were scored. The *p* value was determined by unpaired *t*-tests. Scale bar is 100 μm. **b** Quantitative RT-PCR analysis of Paneth cell gene expression using total RNA isolated from ileal crypts. Crypts were isolated from six mice of each genotype. The *p* values were determined by unpaired *t*-tests. *p < 0.01 (*n* = 6). **c** Double immunostaining of ileal sections using Ufbp1 and lysozyme antibodies. Scale bar is 50 μm

Intestinal stem cells are responsible for renewal of intestinal epithelial cells including Paneth and goblet cells. It remained unclear whether loss of exocrine lineage in *Ufbp1*- or *Ufl1*-deficient intestine is caused by impaired lineage determination of ISCs, or defective development and maturation of Paneth and goblet cells, or both. To address this question, we took advantage of Paneth cell-specific Cre transgenic mouse line in which Cre expression is under the control of defensin α6 (D6) promoter^6^, and created Paneth cell-specific knockout mice of *Ufbp1*. As shown in Fig. 3a, Paneth cell-specific ablation of *Ufbp1* led to fewer Paneth cells in the small intestine, even though the reduction was not as dramatic as in *Ufbp1*^∆/∆IEC^ mice (Figs. 2d and 3a). The decrease of Paneth cells was further confirmed by quantitative RT-PCR analysis of Paneth cell-specific gene expression. Expression of lysozyme and defensin α1 was significantly reduced to half of wild-type level, while expression of chromogranin A (enteroendocrine cell marker), mucin 2 (goblet cell marker) and intestinal alkaline phosphatase (enterocyte marker) remained unchanged in Paneth cell-specific *Ufbp1* KO mice (Fig. 3b). Although the role of Ufbp1 in ISCs remains to be further defined, this result strongly suggests a cell-autonomous role of Ufbp1 in Paneth cell development and maturation after lineage commitment. The difference between two *Ufbp1* KO mouse models was probably due to variable penetrance of Cre transgene expression and efficiency of Cre-mediated deletion of *Ufbp1* allele. This explanation was supported by the observation that nearly all lysozyme-positive Paneth cells in *Ufl1*^f/f^:D6-Cre crypts were also Ufbp1-positive (Fig. 3c).

### *Ufbp1*^∆/∆IEC^ mice had altered fecal microbiota and were susceptible to experimentally induced colitis

**Fig. 4:**
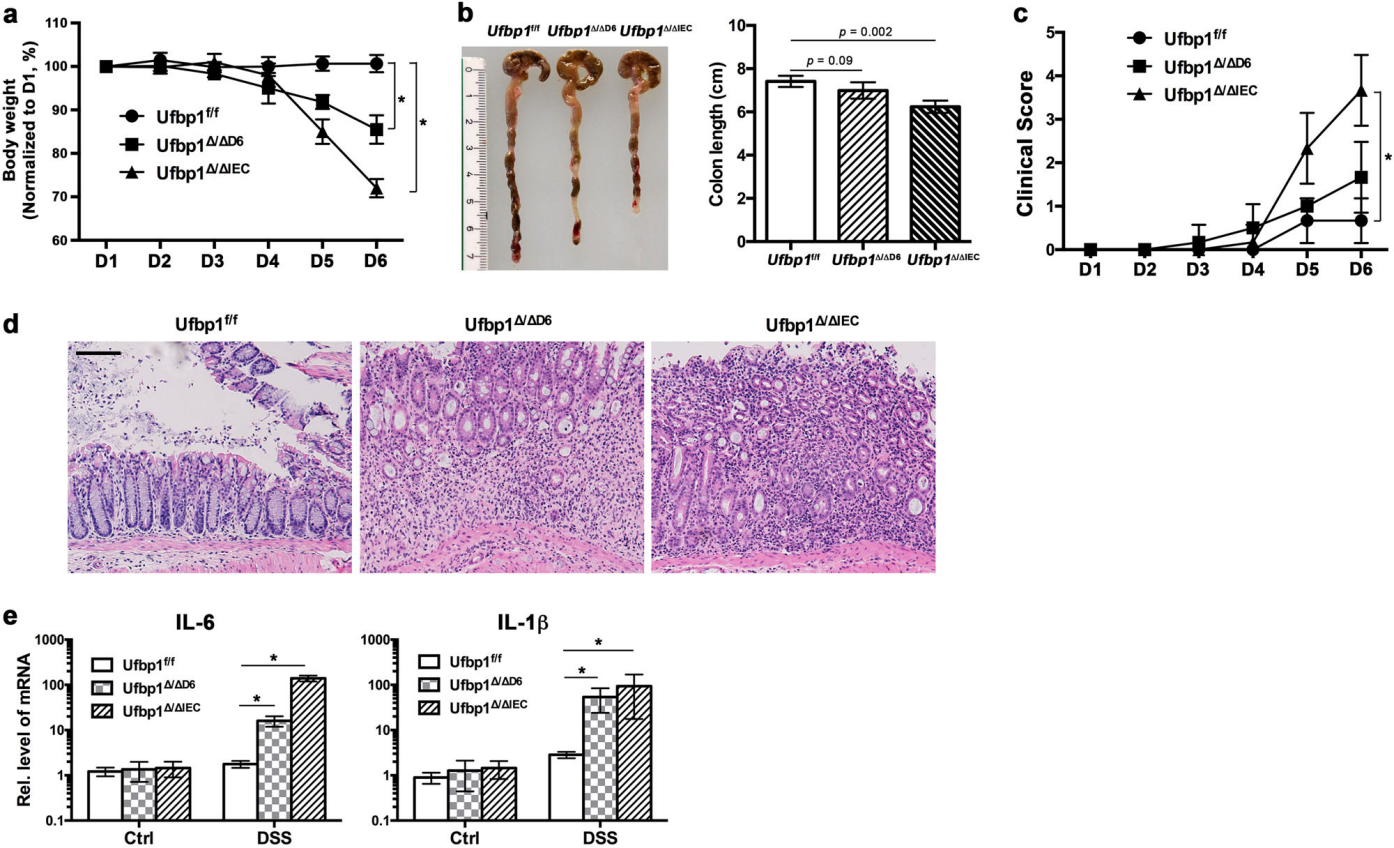
*Ufbp1*^∆/∆IEC^ mice were susceptible to experimentally induced colitis. **a** Weight loss of wild-type and *Ufbp1-*deficient mice during DSS treatment. Six mice of each genotype were treated with 2.5% DSS in drinking water for 5 consecutive days and monitored for weight loss. Weight of individual animal during the treatment was normalized to the initial weight of that animal. Comparing to the wild-type mice that showed very little weight loss during DSS treatment, both IEC- and Paneth cell-specific *Ufbp1-*deficient mice exhibited significant weight loss at day 6. The *p* values were determined by unpaired *t*-tests. *p < 0.01 (*n* = 6). **b** Colon length was reduced in *Ufbp1-*deficient mice after DSS treatment. The *p* values were determined by unpaired *t*-tests (*n* = 6). **c** Clinical scores of diarrhea and anal appearance of DSS-treated mice. Diarrhea: 0 = no diarrhea, 1 = soft but formed solid stool, 2 = very soft stool, 3 = liquid stool, 4 = dysenteric diarrhea; Anal appearance: 0 = normal, 1 = slightly reddish and swelling, 2 = bleeding. The *p* values were determined by unpaired *t*-tests. *p < 0.01 (*n* = 6). **d** H&E staining of colonic sections of wild-type and *Ufbp1-*deficient mice after DSS treatment. In contrast to the relatively normal histology of colonic epithelium in the wild-type mice, *Ufbp1-*deficient colon exhibited severe damages, including loss of basal crypts, massive infiltration of immune cells and destruction of mucosa layer. Scale bar is 100 μm. **e** Elevated expression of inflammatory cytokines in *Ufbp1-*deficient mice after DSS treatment. Total colonic RNA was subjected to quantitative RT-PCR analysis. The *p* values were determined by unpaired *t*-tests. *p < 0.001 (*n* = 6)

It has been shown that dysfunction of Paneth cells in mice leads to dysbiotic microbiota and loss of intestinal homeostasis, thereby conferring susceptibility to experimentally induced colitis^2,3,6,39,40^. We first examined the presence of a few bacterial genera in fecal pellets of *Ufbp1*-deficient mice using 16s rRNA gene-based profiling. As shown in Supplementary Fig. S2, *Ufbp1* deficiency led to alteration of the fecal microbiota: significant decrease of *Bifidobacterium* and increase of *Prevotellacae*. At the age of 8–10 weeks, *Ufbp1*^∆/∆IEC^ intestine did not exhibit signs of inflammation and colitis such as increased lymphocyte infiltration and elevated expression of inflammatory cytokines (see HE staining of intestinal sections in Fig. 2d, e, and the RT-PCR result in Fig. 4e). To address whether *Ufbp1* deficiency predisposes susceptibility to inflammatory colitis, we further tested both IEC- and Paneth cell-specific *Ufbp1*-deficient mice in the DSS (dextran sulfate sodium)-induced colitis model. In comparison to wild-type mice, *Ufbp1*^∆/∆IEC^ mice exhibited accelerated weight loss (Fig. 4a), colon shrinkage (Fig. 4b), deteriorated clinical scores (Fig. 4c), severe epithelial damage including significant loss of basal crypts and massive infiltration of immune cells (Fig. 4d), and elevated expression of inflammatory cytokines IL-1β and IL-6 (Fig. 4e). Interestingly, Paneth cell-specific *Ufbp1*-deficient mice *(Ufbp1*^∆/∆D6^) also showed significant weight loss and increased expression of inflammatory cytokines even though these animals exhibited modest colon shrinkage and worsening of clinical scores (Fig. 4c–e). Taken together, these results suggest that Ufbp1 has a critical role in preventing inflammatory colitis.

### *Ufbp1* deficiency promoted cell death of IECs

**Fig. 5:**
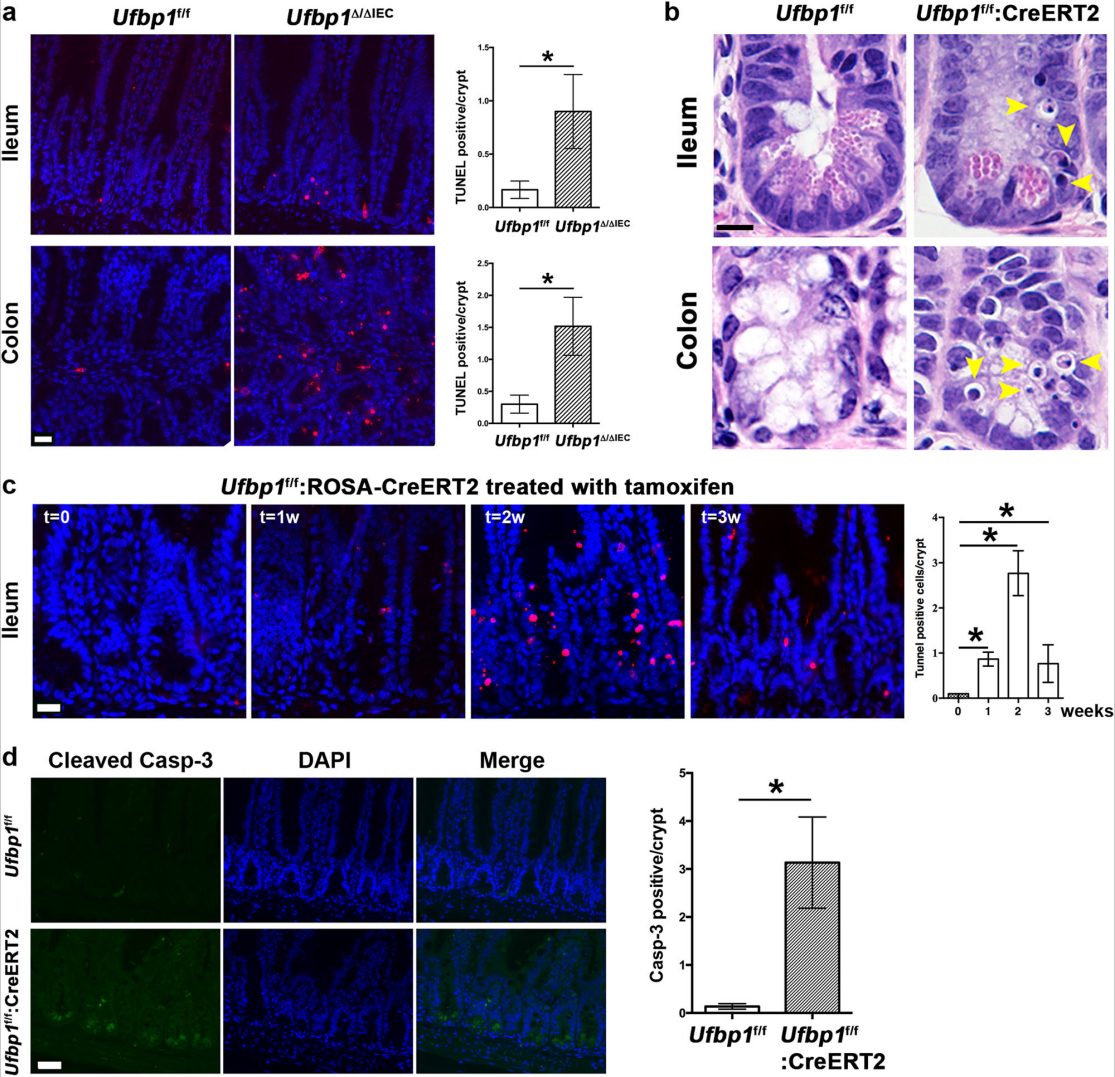
*Ufbp1* deficiency caused cell death of IECs. **a** TUNEL staining of ileal and colonic sections from wild-type and *Ufbp1*^∆/∆IEC^ mice. At least 100 crypts were counted from 6 mice of each genotype in a double blinded manner. Average numbers of TUNEL positive cells per crypt were scored. The *p* values were determined by unpaired *t*-tests. *p < 0.001 (*n* = 6). Scale bar is 20 μm. **b** H&E staining of intestinal sections from the mice with tamoxifen-induced acute deletion of *Ufbp1*. Pyknotic cells were marked by arrowheads. Scale bar is 5 μm. **c** The time course of cell death in the mice with acute deletion of *Ufbp1*. Average numbers of TUNEL positive cells per crypt were scored from 6 mice of each genotype, and at least 100 crypts were counted in a double blinded manner. The *p* values were determined by unpaired *t*-tests. *p < 0.001 (*n* = 6). Scale bar is 20 μm. **d** Immunostaining of cleaved caspase-3 in the ileal sections of wild-type and *Ufbp1*-deficient mice. Ileal sections were from the mice that were treated with tamoxifen for two weeks. At least 100 crypts were counted from 6 mice of each genotype in a double blinded manner. Average numbers of caspase-3 positive cells per crypt were scored. The *p* values were determined by unpaired *t*-tests. *p < 0.001 (*n* = 6). Scale bar is 20 μm

One possible cause for loss of exocrine lineage in *Ufbp1-*deficient intestine is accelerated cell death of exocrine cells. As assessed by TUNEL staining, a substantial increase of cell death was observed in *Ufbp1*^∆/∆IEC^ crypts (Fig. 5a). To better characterize cell death process, we examined *Ufbp1*^*f/f*^:ROSA-CreERT2 mice in which *Ufbp1* was acutely depleted by tamoxifen (TAM) administration. Acute ablation of *Ufbp1* led to marked increase of dying cells with pyknotic nuclei in the crypts, and the dying cells appeared to be Paneth and goblet cells (Fig. 5b). The peak of cell death usually occurred at 2 weeks after initiation of tamoxifen treatment (Fig. 5c). Active caspase-3 positivity was observed in *Ufbp1-*deficient crypts, indicating that apoptosis may be the major form of cell death (Fig. 5d). Taken together, our results suggest that Ufbp1 is crucial for survival of Paneth and goblet cells.

### Depletion of Ufbp1 resulted in activation of the UPR and cell death program in IECs

**Fig. 6:**
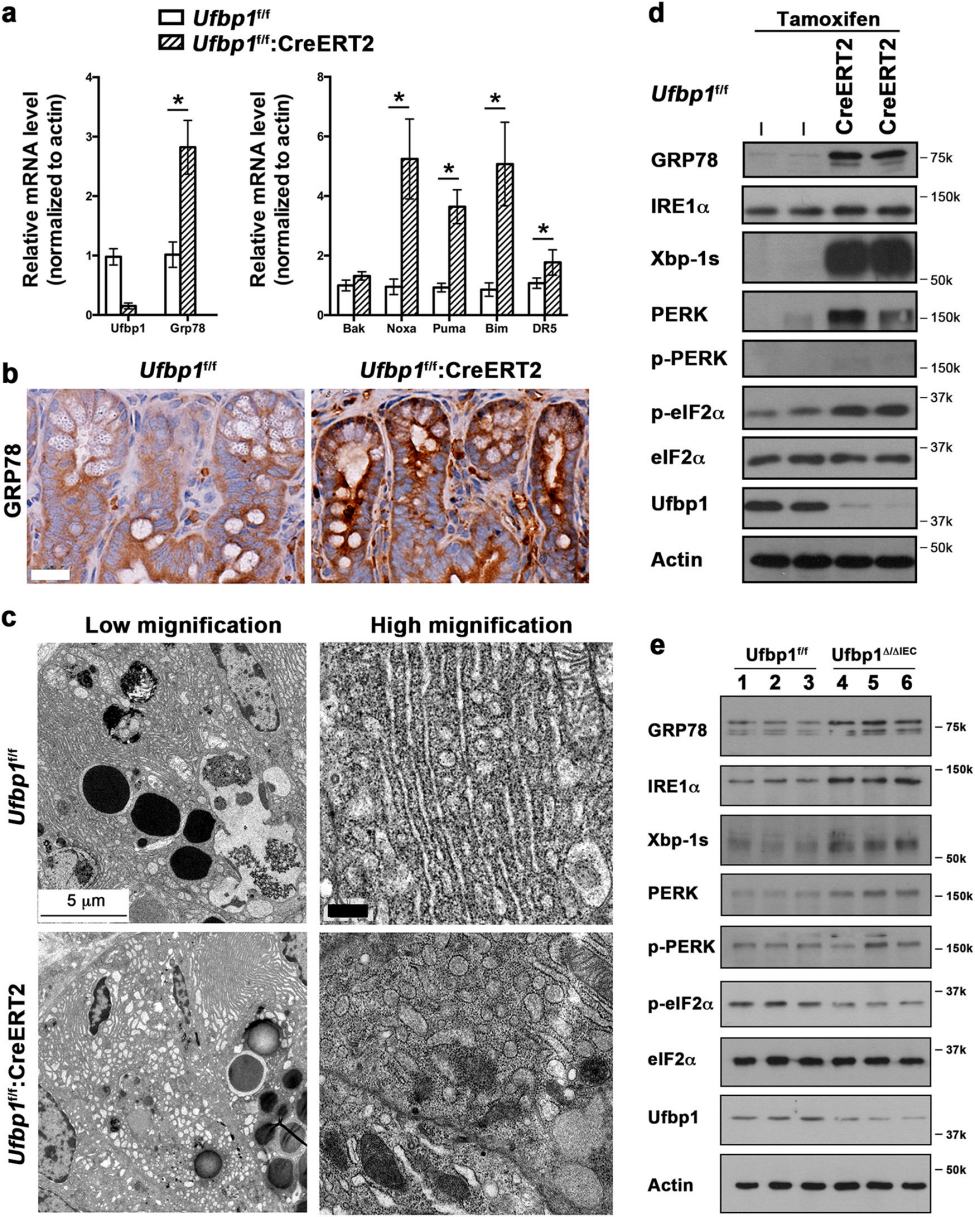
Activation of the UPR and cell death program in *Ufbp1-*deficient IECs. **a** Upregulation of *Grp78* and cell death genes induced by acute ablation of Ufbp1. Mice were treated with tamoxifen for 5 days and then killed at 2 weeks after initial treatment. Total RNA was isolated from ileal crypts and subject to quantitative RT-PCR analysis. The *p* values were determined by unpaired *t*-tests. *p < 0.05 (*n* = 6). **b** Immunohistochemistry of Grp78 protein in the small intestinal sections. Scale bar is 20 μm. **c** Representative EM micrographs of Paneth cells in wild-type and tamoxifen-treated *Ufbp1* floxed mice (1-week treatment). Swelling and vacuolation of the ER structure was observed in *Ufbp1-*deficient Paneth cells. Scale bar is 5 μm for low magnification, and 0.5 μm for high magnification. **d** Activation of UPR pathways in the mice with acute deletion of *Ufbp1*. **e** UPR activation in the *Ufbp1*^∆/∆IEC^ intestine. Crypts were isolated, lysed, and subjected to immunoblotting of specific antibodies as indicated

A previous study indicated that depletion of the Ufm1 components rendered pancreatic β-cells susceptible to ER stress-induced apoptosis^41^. We have shown that the Ufm1 system is upregulated by ER stress^42^, and ablation of either *Ufl1* or *Ufbp1* results in elevated ER stress and activation of UPR in hematopoietic cells^21,22^. Therefore, the Ufm1 system appears to be crucial for ER homeostasis and cellular response to ER stress. In the intestine, acute deletion of *Ufbp1* (*Ufbp1*^*f/f*^:ROSA-CreERT2 treated with tamoxifen) led to upregulation of *Grp78* and a set of cell death genes (Fig. 6a). Elevation of ER stress, indicated by increased staining of Grp78, appeared to occur in all types of IECs (Fig. 6b). In Paneth cells, *Ufbp1* deficiency caused swelling of the ER network (Fig. 6c) and vacuolation of some ER structure (Fig. 6c, high magnification). We further examined activation of downstream signaling branches of the UPR. As shown in Fig. 6d, e, either acute or IEC-specific ablation of *Ufbp1* caused slight increase of IRE1α protein and significant accumulation of Xbp-1s, indicating that the IRE1-Xbp-1 branch of UPR was robustly activated by *Ufbp1* deficiency (Fig. 6d, e). We also observed a substantial increase of total and active PERK in *Ufbp1* knockout IECs, while phosphorylation of eIF2α was slightly increased in the acute deletion model (*Ufbp1*^*f/f*^:ROSA-CreERT2 mice treated with tamoxifen, Fig. 6d) but not significantly changed in the IEC-specific knockout model (Fig. 6e). Being consistent with our previous study, this result demonstrates the crucial role of the ufmylation pathway in modulating ER homeostasis.

### Chemical chaperone TUDCA alleviates loss of *Ufbp1*-deficient Paneth cells

**Fig. 7:**
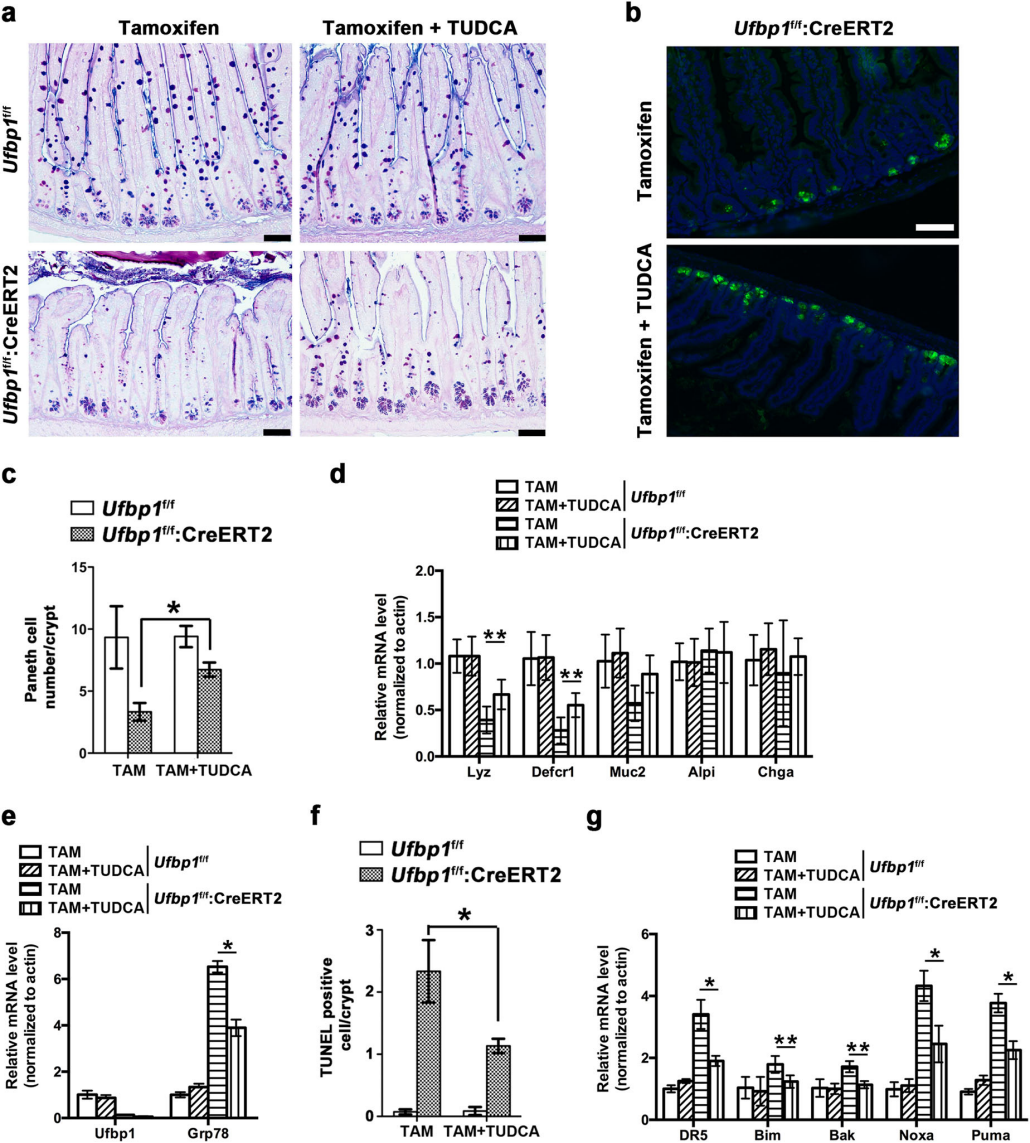
TUDCA prevents loss of Paneth cells in *Ufbp1-*deficient mice. **a** Alcian/PAS staining of ileal sections. 8 mice in each genotype (8- to 10-week male) were divided into two groups: one group were gavaged with saline and another with TUDCA (150 mg/kg) daily for 14 days. Tamoxifen (TAM, 75 mg/kg) was injected i.p. at day 2 for 5 days. Mice were killed at day 15. Scale bar is 100 μm. **b** Lysozyme staining of ileal sections of TAM-treated mice with or without TUDAC. Scale bar is 100 μm. **c** Quantitation of Paneth cell numbers in each group of mice. At least 100 crypts were counted from 4 mice of each genotype in a double blinded manner. The *p* values were determined by unpaired *t*-tests. *p < 0.01 (*n* = 4). **d** Quantitative RT-PCR analysis of Paneth cell gene expression. Total RNAs were isolated from the intestine and used for the analysis. The *p* values were determined by unpaired *t*-tests. **p < 0.05 (*n* = 4). *Lyz* lysozyme; *Defcr1* defensin α1; *Alpi* alkaline phosphatase; *Chga* Chromogranin A; and *Muc2* mucin 2. **e** Quantitative RT-PCR analysis of Grp78 expression in the intestine. Total RNAs were isolated from the intestine and used for the analysis. The *p* values were determined by unpaired *t*-tests. *p < 0.01 (n = 4). **f** Quantitation of TUNEL positive cells. At least 100 crypts were counted from 4 mice of each genotype in a double blinded manner. The *p* values were determined by unpaired *t*-tests. *p < 0.01 (*n* = 4). **g** Quantitative RT-PCR analysis of cell death gene expression. The *p* values were determined by unpaired *t*-tests. *p < 0.01 and **p < 0.05 (*n* = 4)

To further evaluate the consequence of elevated ER stress and UPR activation in *Ufbp1-*deficient IECs, we tested the effect of tauroursodeoxycholic acid (TUDCA), a natural bile salt that mitigates ER stress, in the acute knockout model^43–45^. As indicated by the Paneth cell number and gene expression, TUDCA treatment significantly alleviated loss of Paneth cells in *Ufbp1-*deficient crypts (Fig. 7a–d). Moreover, TUDCA partially suppressed *Ufbp1* deletion-induced upregulation of *Grp78* (Fig. 7e), cell death (Fig. 7f) and elevated expression of cell death genes (Fig. 7g). Together, these results suggest that elevated ER stress contributes to loss of *Ufbp1*-deficient Paneth cells.

## Discussion

Recent genetic studies using knockout mouse models have established the indispensable role of the ufmylation pathway in embryonic development and hematopoiesis^20–22^. Yet its physiological functions in other tissues and organs have not been defined. In this study, we report that both Ufl1 and Ufbp1, two key components of a Ufm1-specific E3 ligase, are highly expressed in intestinal exocrine cells and essential for their development and survival. Acute ablation of *Ufl1* resulted in significant loss of Paneth and goblet cells, while IEC-specific *Ufbp1* knockout mice exhibited the same phenotype. Loss of Paneth cells in *Ufbp1*^∆/∆IEC^ mice resulted in increased susceptibility to DSS-induced colitis. At the cellular and molecular levels, *Ufbp1* deficiency led to elevated ER stress and activation of UPR, which in turn caused apoptosis of Paneth and goblet cells. Administration of a small chemical chaperone TUDCA partially prevented loss of Paneth cells in *Ufbp1*-deficient crypts. Taken together, our results have demonstrated a pivotal role of Ufl1 and Ufbp1 in development and survival of exocrine cell lineage and maintenance of intestinal homeostasis.

Intestinal exocrine cells including Paneth and goblet cells are responsible for production and secretion of a large amount of proteins that are essential for intestinal homeostasis and mucosal immunity. At the cellular level, both Paneth and goblet cells contain a rich rough ER network to cope with the demand for protein synthesis, modification, folding and secretion. Therefore, these cells are much more vulnerable to any perturbation of ER homeostasis and elevation of ER stress, a condition which is usually a result of accumulation of unfolded and misfolded proteins in the ER. Many environmental factors, such as oxidative stress, viral infection, inflammation and nutrient deficiency, often cause elevation of ER stress in intestinal epithelial cells^46,47^. In response to elevated ER stress, cells activate the UPR, a highly conserved signaling pathway, to facilitate the folding of unfolded or misfolded proteins and restoration of ER homeostasis. Given its important role in maintaining proper ER function, it is not surprising that intestinal exocrine cells, such as Paneth and goblet cells whose main function is to synthesize and secrete a large quantity of proteins, are highly dependent on the functional UPR. Any disturbance or disruption of the UPR may cause severe reduction or even loss of their secretory capacity, which in turn leads to dysbiotic intestine and inflammatory diseases. Consistent with this notion, IBD patients were reported to have increased ER stress in ileul and/or colonic epithelia^48–50^. Furthermore, genome-wide association study (GWAS) and deep sequencing of IBD patients has identified several UPR-related genes as IBD susceptibility loci. Rare variants of *Xbp-1* were identified as a risk locus for both ulcerative colitis and Crohn’s disease, and its deficiency in mouse intestine led to complete loss of Paneth cells and partial loss of goblet cells, thereby resulted in increased susceptibility to DSS-induced colitis^51^. In addition, key UPR molecules such as IRE1 and eIF2α also have a critical role in epithelial integrity and intestinal homeostasis. *IRE1*β knockout caused aberrant accumulation of mucin 2 mRNA and protein, resulting in ER distension, elevated ER stress and barrier function defect^52^. IEC-specific ablation of IRE1α leads to loss of goblet cells and spontaneous colitis, and *IRE1*α^*−/−*^ mice are more susceptible to DSS-induced colitis and ER stress-related apoptosis^53^. Phosphorylation of eIF2α, a key step in the UPR to attenuate protein translation, is essential for Paneth cell function, and the mice with loss of eIF2α phosphorylation are more susceptible to *Salmonella* infection and experimentally induced colitis^54^. Deficiency of AGR2, an ER-resided disulfide isomerase, also disrupted intestinal tissue homeostasis and caused development of spontaneous ileitis and colitis in mice^55,56^.

Our current study has identified the Ufm1 conjugation system, a metazoan-specific ubiquitination-like system, as a novel regulator of intestinal homeostasis. Mouse genetic studies have demonstrated its indispensable role in embryonic development, yet the working mechanism remains poorly understood at the cellular level. Emerging evidence from recent studies suggest its crucial role in maintaining ER homeostasis. Ufl1 and Ufbp1 are ER-anchored proteins, and other components of ufmylation are also mainly associated with the ER^14,16^. In addition to ubiquitous expression of ufmylation-related genes in many cell types and tissues, we found that the components of the Ufm1 system, including Ufl1 and Ufbp1, were highly expressed in intestinal exocrine cells that contain very rich ER structure (Fig. 1a). Additionally, Ufm1 was reported to be a target of secretory cell-specific transcription factor MIST1^57^, while several ufmylation-related genes are transcriptionally regulated by Xbp-1, a key transcription factor of the UPR^42^. The expression profile of these ufmylation genes is consistent with their functional role in the cells with rich ER structure. Deficiency of ufmylation genes such as *Uba5* and *Ufm1* led to accumulation and expansion of ER membranes^30,42^. Furthermore, overexpression of the components of ufmylation protects pancreatic beta cells and macrophages from ER stress-induced cell death^41,58^. Our previous and current studies have demonstrated that knockout of either *Ufl1* or *Ufbp1* resulted in elevated ER stress and activation of the UPR in various types of cells, including hematopoietic and intestinal cells (^21,22^ and the results in this manuscript). Intriguingly, our findings were consistent with the results of recent studies conducted by DeJesus et al.^59^ and Terai et al.^60^ In genome-wide CRISPR/cas9 screenings, knockout of the ufmylation genes triggered the UPR activation in multiple cancer cell lines^59,60^. Taken together, the recent findings strongly suggest that the ufmylation pathway is a novel mechanism to regulate ER homeostasis and function, and deficiency of ufmylation may cause perturbation of ER homeostasis and elevated ER stress that leads to apoptosis of exocrine cells.

The next question is how ufmylation is engaged in the maintenance of ER homeostasis. One possible scenario is that the ufmylation pathway may modulate the activity of the key UPR player(s) in a direct manner. Liu et al.^61^ reported that Ufbp1 can directly interact and stabilize IRE1α, and knockdown of Ufbp1 resulted in IRE1α degradation and attenuation of the IRE1α-Xbp1 signaling, which in turn activated the PERK signaling. However, in contrast to their findings, we found that *Ufbp1* deficiency led to IRE1α upregulation and robust Xbp-1 activation (Fig. 6). Our finding is in agreement with the study by Terai et al.^60^, showing that CRISPR/Cas9-mediated knockout of ufmylation genes in lung cancer cells specifically activated the IRE1α-Xbp1 branch. Apparently, more work is needed to establish the molecular link(s), either direct or indirect, between the ufmylation and UPR pathways. Another scenario is that the ufmylation pathway may be involved in controlling ER-related functions such as protein synthesis, modification, folding and secretion, and its deficiency may lead to accumulation of unfolded or misfolded protein, thereby resulting in ER stress elevation and UPR activation. Recently, Simsek et al.^62^ reported that Ufl1 was one of ribosome-associated proteins, and several ribosomal subunits and elongation factor eIF6 were potential Ufm1 targets. This finding indicates a potential role of ufmylation in control of protein translation. Interestingly enough, protein translation inhibitor cycloheximide can promote Ufm1 conjugation in tissue culture cells^41,42^. We speculate that the ufmylation pathway may represent a novel generic mechanism to co-ordinate protein translation and protein quality control in the ER. Further study, especially identification and characterization of Ufm1 targets and their interacting proteins, is needed to test this hypothesis. In summary, our current work uncovered a crucial link between the ufmylation pathway and intestinal homeostasis. These findings may provide a basis for identification of novel therapeutic targets for treatment of IBD and other gastrointestinal diseases.

## Materials and methods

### Ufl1 and Ufbp1 knockout mice and experimental procedures

*Ufl1* and *Ufbp1* knockout mouse lines and genotyping methods were described in our previous reports^21,22^. Villin-Cre transgenic mouse line was originally from Dr. Sylvie Robine’s laboratory^38^. Paneth cell-specific Defa6-Cre transgenic line was obtained from Dr. Richard Blumberg’s laboratory^6^. All mice were maintained in C57BL/6 background. Cre-mediated acute deletion of *Ufl1* or *Ufbp1* was induced by tamoxifen administration. Tamoxifen (20 mg/ml in corn oil, Sigma, St. Louis, MO) was administered by 5-day intraperitoneal (IP) injection with an approximate dose of 75 mg tamoxifen/kg body weight. At two days prior to tamoxifen injection, tauroursodeoxycholic acid (TUDCA, 150 mg/kg, Cayman Chemical, Ann Arbor, MI) was administrated daily by gavage, and the treatment was then continued through the course of experiment. Colitis-grade dextran sulfate sodium (DSS, MW 36,000–50,000) was purchased from MP Biomedicals (Santa Ana, CA). Mice were fed with 2.5–5% DSS in the drinking water for 5 days. Mice were housed in the animal facility at Augusta University, and all animal procedures were approved by IACUC of Augusta University.

### Isolation and in vitro organoid culture of intestinal crypts

Crypts were isolated from the ileum of small intestine according to Sato et al.^63^ with minor modifications. Briefly, the ileum of small intestine was harvested and opened lengthwise, and then washed multiple times with cold PBS. After removal of the villi with a cover glass, the intestine was cut into 2–3 large pieces, and then washed with cold PBS (10–20 ml) for more than 10 times. Subsequently the fragments were incubated in 25 ml of 2 mM EDTA on ice for 15 min with a gentle shaking. After removal of the supernatant, the tissue was washed with cold PBS and then pipetted up and down 3–5 times. Released crypts were passed through a 70 μM cell strainer and collected by 3-min centrifugation at 100×*g*. Isolated crypts were counted and resuspended in Growth Factor Reduced Matrigel (Corning Life sciences, Corning, NY) and cultured in the complete medium: Advanced DMEM/F12 supplemented with 1×B27, 1×N2 (Thermo Fisher Scientific, Waltham, MA), 1 mM *N*-acetyle-l-cysteine, HEPES (10 mM, pH 7.4), murine EGF (50 ng/ml, PeproTech, Rocky Hill, NJ), murine Noggin (100 ng/ml, PeproTech), 1/10th volume of R-Spondin-1 conditional medium (Trevigen, Gaitherburg, MD), 1×GlutaMax, 1×Penicillin/ Streptomycin (Thermo Fisher Scientific), and 100 μg/ml Normocin (Invivogen, San Diego, CA),

### Histology, immunohistochemistry, immunofluorescent staining, and immunoblotting

HE and PAS/Alcian blue staining was performed by the Histology core of Augusta University, while Transmission Electron Microscope (TEM) was conducted by the EM core of Augusta University according to the standard procedures^22^. Immunohistochemistry, immunofluorescent staining and Immunoblottings were performed as described previously^22^. Bright field and Epifluorescence images were obtained using Zeiss Observer D1 with AxioVision 4.8 software (Carl Zeiss Microscopy GmbH, Jena, Germany) and Keyence BZ-X700 fluorescent microscope with its corresponding software (Keyence America, Itasca, IL, USA).

The antibodies used in this study included: Ufl1 and Ufbp1 rat polyclonal antibodies (Li lab), Ufbp1 rabbit polyclonal antibody (21445–1-AP, Proteintech, Rosemont, IL), eIF2α (#5324, Cell Signaling, Danvers, MA), phospho-S51-eIF2α (#3398, Cell Signaling), Lysozyme (A0099, Agilent Dako, Santa Clara, CA), Grp78/Bip (#3177, Cell Signaling), IRE1α (#3294, Cell Signaling), Xbp-1s (#619502, Poly6195, BioLegend, San Diego, CA), PERK (#3192, Cell Signaling), phospho-PERK (Thr980) (#3197, Cell Signaling), cleaved caspase-3 (Asp175) (#9664, Cell Signaling), Chromogranin A (Ab15160, Abcam, Cambridge, MA) and β-Actin (#3700, Cell Signaling). All affinity-purified and species-specific HRP- and fluorophore-conjugated secondary antibodies were obtained from Jackson ImmunoResearch (West Grove, PA).

### TUNEL staining

TUNEL staining was performed using in situ cell death detection kit (TMR Red, Roche, Basel, Switzerland) according to the manufacturer’s instruction.

### Quantification of gut microbiota using 16s rRNA profiling

Quantification of gut microbiota was performed according to Sivaprakasm et al.^64^ Feces (~100 mg) were suspended with 710 μl of disruption buffer (200 mm NaCl, 200 mm Tris pH 8.0, 20 mm EDTA pH 8.0 and 6% SDS) and 500 μl of phenol/chloroform/isoamyl alcohol, pH 8.0 inside tubes containing Zirconium beads (0.1 mm diameter, Benchmark Scientific, Edison, NJ, USA). The mixture was homogenized using a Beadbeater (BioSpec, Bartlesville, OK, USA) for 2 cycles of 2 min each. Sample was centrifuged at 7000 g for 3 min and aqueous phase was collected and a second round of phenol/chloroform/isoamyl extraction was performed. DNA from the clear aqueous phase was precipitated using sodium acetate and isopropanol. Dried DNA pellet was dissolved in TE buffer. Quantitative PCR using group-specific PCR primers were used to quantify relative abundance of different groups to the total gut microbiota.

**Table:** 

Target	Forward primer	Reverse primer
All bacteria	ACTCCTACGGGAGGCAGCAGT	GTATTACCGCGGCTGCTGGCAC
*Bifidobacterium*	TCGCGTCYGGTGTGAAAG	CCACATCCAGCRTCCAC
*Prevotellaceae*	CCAGCCAAGTAGCGTGCA	TGGACCTTCCGTATTACC

### Quantitative real-time PCR

Total RNA from each sample was isolated with the GeneJET RNA Purification Kit (Thermo Fisher Scientific) or TRIzol reagent (Thermo Fisher Scientific), and then reversely transcribed using AMV reverse transcriptase and random primers according to the manufacturer’s instruction (Thermo Fisher Scientific). Quantitative RT-PCR was performed using the iTaq Universal SYBR Green Supermix kit (BIO-RAD, Hercules, CA) with 40 cycles of 95 °C for 15 s and 60 °C for 1 min on StepOnePlus Real-Time PCR System (Thermo Fisher Scientific). The results were analyzed by StepOne Software (Version 2.1, Life Technologies). Relative expression level of each transcript was normalized to murine beta-actin by using the 2^(−delta delta Ct)^ method. The following is the list of primers used in this study:

**Table:** 

Gene (mouse)	Forward primer	Reverse primer
*Actin*	GACCTCTATGCCAACACAGT	AGTACTTGCGCTCAGGAGGA
*Ufl1 (exon7-specific)*	CCAGTGAACTCTTTGGTTTCAA	CCATTCTGCCTGAAAAAGGA
*Ufbp1 (exon3,4-specific)*	GAAGCCAGCAGAAGTTCACC	GAAGCCGTTCCTCTTCCTTC
*Grp78/Bip*	ACTTGGGGACCACCTATTCCT	ATCGCCAATCAGACGCTCC
*Bak*	AAAATGGCATCTGGACAAGG	AAGATGCTGTTGGGTTCCAG
*Noxa*	GGCAGAGCTACCACCTGAGT	TTGAGCACACTCGTCCTTCA
*Puma*	GCCCAGCAGCACTTAGAGTC	TGTCGATGCTGCTCTTCTTG
*Bim*	TGCAGAGGATGATTGCTGAC	AGTACTTGCGCTCAGGAGGA
*DR5*	TGACTACACCAGCCATTCCA	AGTTCCTCTTCCCCGTCAGT
*Lysozyme 2*	ATGGAATGGCTGGCTACTATGG	ACCAGTATCGGCTATTGATCTGA
*Bim*	TGCAGAGGATGATTGCTGAC	AGTACTTGCGCTCAGGAGGA
*Defcr1*	AAGAGACTAAAACTGAGGAGCAGC	CGACAGCAGAGCGTGTA
*Defcr5*	AGGCTGATCCTATCCACAAAACAG	TGAAGAGCAGACCCTTCTTGGC
*Intestinal alkaline phosphatase*	CACAGCTTACCTGGCACTGA	GGTCTCTGACGACAGGGGTA
*Chromogranin A*	CCAATACCCAATCACCAACC	TTGTAGCCTGCATGGAAGTG
*Mucin 2*	GCCTGTTTGATAGCTGCTATGTGCC	GTTCCGCCAGTCAATGCAGACAC

### Statistical analysis

All statistical analyses were performed using Graph Prism software. *p* values were determined by unpaired *t*-tests between two set of data. A *p* value <0.05 was considered to be significant.

## Electronic supplementary material

Supplementary Information

## Electronic supplementary material

**Supplementary Information** accompanies the paper at (10.1038/s41421-018-0070-x).
