# Supplementary material for: Indispensable role of the Ubiquitin-fold modifier 1-specific E3 ligase in maintaining intestinal homeostasis and controlling gut inflammation
Source: Cell Discov. 2019 Jan 29;5:7. doi: 10.1038/s41421-018-0070-x (PMC6349939; doi:10.1038/s41421-018-0070-x)

Supplementary Fig. S1a. Expression of Paneth cell-specific genes in wild-type and *Ufbp1*$^{\Delta/\Delta IEC}$ mice. Quantitative RT-PCR analysis was performed using total RNA isolated from ileal section of small intestine (3 mice for each genotype). * p < 0.01. **S1b.** Chromogranin A (CgA)-positive enteroendocrine cells in in wild-type and *Ufbp1*$^{\Delta/\Delta IEC}$ mice.

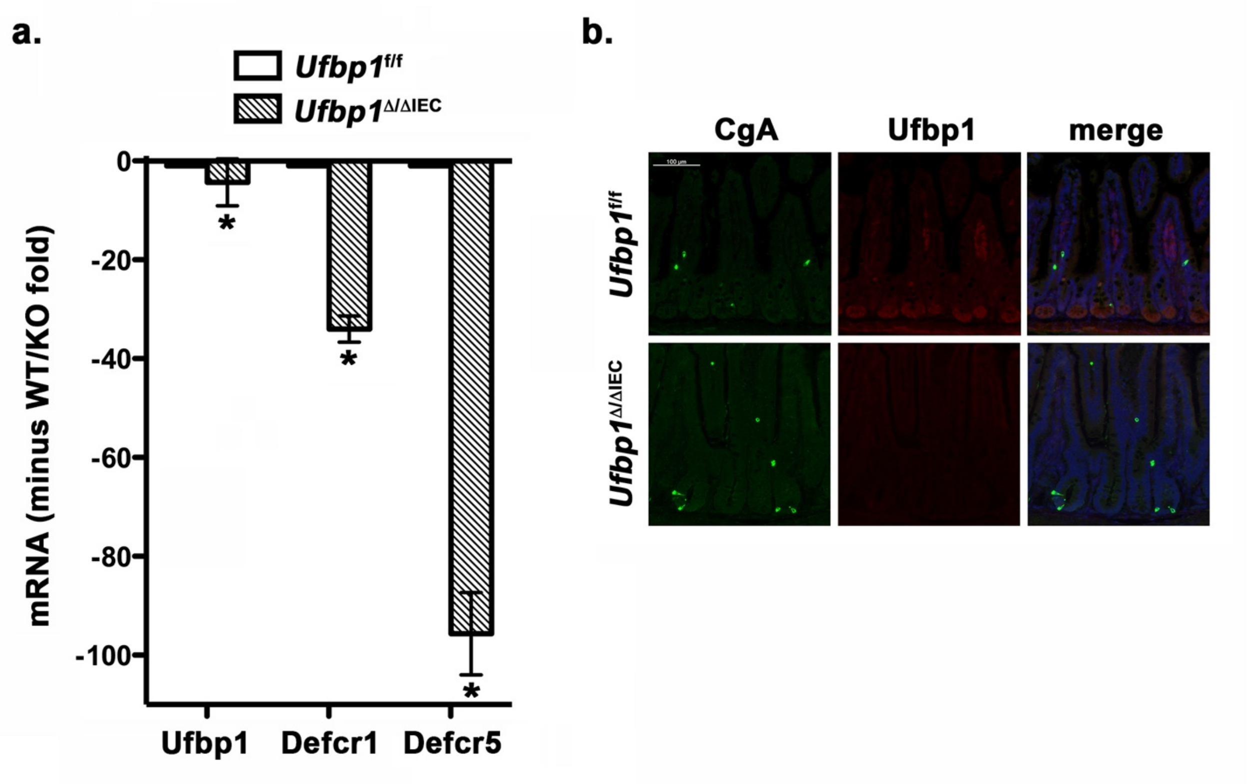

Supplementary Fig. S2 Alteration of intestinal bacterial genera in *Ufbp1* deficient mice. Fecal DNA was isolated from fecal pellets (8 mice for each genotype), and subjected to quantitative PCR using 16s rRNA-specific primers. * p <0.001.

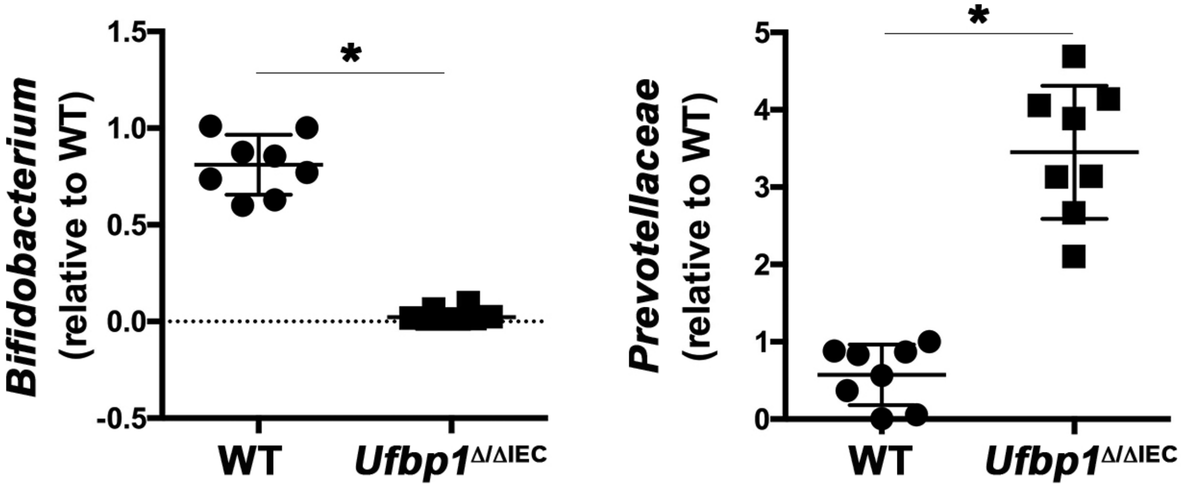

Supplement: Supplementary file 1 — Supplementary Information [file 41421_2018_70_MOESM1_ESM.pdf]
